# Risk of Depressive Disorder following Non-Alcoholic Cirrhosis: A Nationwide Population-Based Study

**DOI:** 10.1371/journal.pone.0088721

**Published:** 2014-02-12

**Authors:** Chin-Lin Perng, Cheng-Che Shen, Li-Yu Hu, Chiu-Mei Yeh, Mu-Hong Chen, Chia-Fen Tsai, Huey-Ling Chiang, Yi-Ping Hung, Vincent Yi-Fong Su, Yu-Wen Hu, Tung-Ping Su, Pan-Ming Chen, Jeng-Hsiu Hung, Chia-Jen Liu, Min-Wei Huang

**Affiliations:** 1 Division of Gastroenterology, Department of Medicine, Taipei Veterans General Hospital, Taipei, Taiwan; 2 School of Medicine, National Yang-Ming University, Taipei, Taiwan; 3 Department of Psychiatry, Chiayi Branch, Taichung Veterans General Hospital, Chiayi, Taiwan; 4 Department of Information Magagement, National Chung-Cheng University, Chiayi, Taiwan; 5 Department of Psychiatry, Kaohsiung Veterans General Hospital, Kaohsiung, Taiwan; 6 Department of Family Medicine, Taipei Veterans General Hospital, Taipei, Taiwan; 7 Department of Psychiatry, Taipei Veterans General Hospital, Taipei, Taiwan; 8 Department of Psychiatry, Far Eastern Memorial Hospital, New Taipei City, Taiwan; 9 Department of Psychiatry, National Taiwan University Hospital and College of Medicine, Taipei, Taiwan; 10 Division of Hematology and Oncology, Department of Medicine, Taipei Veterans General Hospital, Taipei, Taiwan; 11 Department of Chest Medicine, Taipei Veterans General Hospital, Taipei, Taiwan; 12 Cancer Center, Taipei Veterans General Hospital, Taipei, Taiwan; 13 Institute of Public Health, National Yang-Ming University, Taipei, Taiwan; 14 Department of Psychiatry, Su-Ao and Yuanshan Branch, Taipei Veterans General Hospital, Taipei, Taiwan; 15 Department of Obstetrics and Gynecology, Taipei Tzu Chi Hospital, Buddhist Tzu Chi Medical Foundation, Taipei, Taiwan; 16 School of Medicine, Tzu Chi University, Hualien, Taiwan; Xi'an Jiaotong Univesity School of Medicine, China

## Abstract

**Background & Aims:**

To evaluate the risk of depressive disorders among non-alcoholic patients by using the Taiwan National Health Insurance Research Database (NHIRD).

**Methods:**

We conducted a retrospective study of a matched cohort of 52 725 participants (10 545 non-alcoholic cirrhotic patients and 42 180 control patients) who were selected from the NHIRD. Patients were observed for a maximum of 11 years to determine the rates of newly onset depressive disorders, and Cox regression was used to identify the risk factors associated with depressive disorders in cirrhotic patients.

**Results:**

During the 11-year follow-up period, 395 (3.75%) non-alcoholic cirrhotic patients and 1 183 (2.80%) control patients were diagnosed with depressive disorders. The incidence risk ratio of depressive disorders between non-alcoholic cirrhotic patients and control patients was 1.76 (95% CI, 1.57–1.98, *P*<.001). After adjusting for age, sex, and comorbidities, non-alcoholic cirrhotic patients were 1.75 times more likely to develop depressive disorders (95% CI, 1.56–1.96, *P*<.001) compared with the control patients. The hazard ratios for patients younger than 60 years old (1.31) and female (1.25) indicated that each is an independent risk factor for depressive disorders in non-alcoholic cirrhotic patients.

**Conclusions:**

The likelihood of developing depressive disorders is greater among non-alcoholic cirrhotic patients than among patients without cirrhosis. Symptoms of depression should be sought in patients with cirrhosis.

## Introduction

Cirrhosis is a consequence of chronic liver disease characterized by the replacement of liver tissue by fibrosis, scar tissue, and regenerative nodules, which leads to loss of liver function. Cirrhosis and chronic liver disease have been leading causes of death in the United States and incur considerable healthcare costs, particularly, costs related to loss of productivity [1].

Some studies have reported a high depression prevalence rate in patients with cirrhosis [2]–[5], and_ENREF_2 a significant correlation was observed among depression and quality of life in cirrhotic patients [5]–[7]. In addition, cirrhotic patients diagnosed with depression were significantly more likely to die while awaiting liver transplantation than were non-depressed cirrhotic patients, independent of the severity of liver disease and its complications [6]. Hence, the depression symptoms should be examined in cirrhotic patients because depression is a modifiable illness that is amenable to treatment.

Although previous studies focusing on depression in cirrhotic patients [2]–[8] have addressed alcoholic cirrhosis, establishing whether the reported depression was related to alcohol or cirrhosis is difficult because alcohol users have been highly associated with psychiatric disorders, especially depressive disorders [9]–[14]. In addition, in these studies, depression was often evaluated using rating scales, such as the Beck depression inventory or the Hamilton depression rating scale, rather than diagnosis by a physician. The real prevalence rate of clinical depressive disorders in cirrhotic patients remains unknown. Furthermore, whether there is an independent risk factor for newly diagnosed depressive disorders in non-alcoholic cirrhotic patients has never been studied.

We performed a population-based retrospective cohort study using data derived from the National Health Insurance (NHI) system in Taiwan. The purpose of our study was to determine whether non-alcoholic cirrhosis was associated with an increased risk of clinical depressive disorders. Independent risk factors for newly diagnosed depressive disorders in the non-alcoholic cirrhotic patients were also identified.

## Materials and Methods

### Data Sources

Instituted in 1995, the NHI program is a mandatory health insurance program that offers comprehensive medical care coverage, including outpatient, inpatient, emergency, and traditional Chinese medicine, to all residents of Taiwan; the coverage rate is as high as 99%.[15] The NHI research database (NHIRD) contains comprehensive information regarding clinical visits, including prescription details and diagnostic codes based on the International Classification of Diseases, ninth revision, Clinical Modification (ICD-9-CM). The NHIRD is managed by the National Health Research Institutes (NHRI) and confidentiality is maintained according to the directives of the Bureau of the NHI. We used the Longitudinal Health Insurance Database 2000 (LHID 2000) as the data source for our study, which is a data set released by the NHRI that contains all original claims data for 1 million randomly selected beneficiaries in the 2000 Registry of Beneficiaries.

### Ethics Statement

The Institutional Review Board of the Taipei Veterans General Hospital approved this study (2013-08-016BC). Written consent from study patients was not obtained because the NHI dataset consists of de-identified secondary data for research purposes, and the Institutional Review Board of Taipei Veterans General Hospital issued a formal written waiver for the need for consent.

### Study Population

Using data extracted from the LHID 2000, we conducted a retrospective cohort study of patients aged 20 years and older who were newly diagnosed with non-alcoholic cirrhosis between January 1, 2000 and December 31, 2009. Non-alcoholic cirrhosis was defined as cirrhosis of the liver without mention of alcohol (ICD-9-CM code: 571.5) and biliary cirrhosis (ICD-9-CM code: 571.6). We excluded patients who were diagnosed with depressive disorders (ICD-9-CM code: 296.2X-296.3X, 300.4, and 311.X) [16], bipolar disorders (ICD-9-CM code: 296.0, 296.1, 296.4, 296.5, 296.6, 296.7, 296.8, 296.80, and 296.89) [17], or alcohol-use disorders (ICD-9-CM codes: V113, 9800, 2650, 2651, 3575, 4255, 3050, 291, 303, and 571.0-571.3) [17] before enrollment. Insurance premiums, calculated according to the beneficiary's total income, were used to estimate monthly income. Monthly income was grouped into low income (monthly income <20,000 New Taiwan Dollar [NTD]), median income (20,000 NTD ≤ monthly income <40,000 NTD), and high income (monthly income ≥ 40,000 NTD). Urbanization was divided into three groups: urban, suburban, and rural. Urbanization and monthly income levels were used to represent socioeconomic status. For each cirrhotic patient included in the final cohort, 4 age-, sex-, common-comorbidities-, urbanization-, monthly-income-, and enrolment-date-matched control patients who were not diagnosed with cirrhosis or a psychiatric disease (ICD-9-CM code: 290-319) were randomly selected from the LHID 2000. We selected control patients by conducting incidence density sampling using computer programming [18]. All cirrhotic and control patients were observed until diagnosed with a depressive disorder according to ICD-9-CM codes (ICD-9-CM code: 296.2X-296.3X, 300.4, and 311.X), death, withdrawal from the NHI system, or December 31, 2010. The primary clinical outcome assessed was psychiatrist-diagnosed depressive disorder.

### Statistical Analyses

The incidence of newly diagnosed depressive disorders in the non-alcoholic cirrhotic and control patients, stratified by gender and age (equal or older than 60 years old or younger than 60 years old) was calculated. Independent *t*-tests and chi-squared tests were used to examine the differences in the demographic characteristics between the cirrhotic and control patients.

A Cox proportional-hazards regression model was used to identify variables that predicted depressive disorder in the non-alcoholic cirrhotic and control patients, and in the non-alcoholic cirrhotic patients only. Control variables, such as age; sex; common comorbidities, including diabetes mellitus, congestive heart failure, chronic kidney disease, autoimmune disease, cerebrovascular disease, coronary artery disease, and malignancy; urbanization; and monthly income were included as covariates in the univariate model. Factors that demonstrated a moderately significant statistical relationship in the univariate analysis (*P*<.1) were entered by forward selection in a multivariate Cox proportional-hazards regression model [19].

The Perl programming language (version 5.12.2) was used to extract and compute data. The Microsoft SQL Server 2005 (Microsoft Corp., Redmond, WA, USA) was used for data linkage, processing, and control sampling. IBM SPSS (version 19.0 for Windows; IBM Corp., New York, NY, USA) and SAS statistical software (version 9.2; SAS Institute Inc., Cary, NC, USA) were used to perform all statistical analyses. The comparisons resulting in a *P*-value of less than .05 were considered to indicate a statistically significant relationship.

## Results

### Participant Selection

The sample comprised 10 545 non-alcoholic cirrhotic patients and 42 180 control patients without depression, among whom 62.6% were male. The median age at enrollment was 59 years (interquartile range [IQR], 47–70 years), and the median follow-up period was 3.90 years (IQR, 1.26–7.30 years) for cirrhotic patients and 5.85 years (IQR, 3.21–8.53 years) for control patients. The comparisons of the demographic and clinical variables between the cirrhotic and control patients are presented in Table 1.

**Table 1:** Baseline Characteristics of Patients with and without Non-alcoholic Cirrhosis.

Demographic data	Patients with non-alcoholic cirrhosis *n* = 10,545		Patients without non-alcoholic cirrhosis *n* = 42,180		*P*-value
	*n*	%	*n*	%	
Age (years) (interquartile range)	59 (47–70)		59 (47–70)		
≥60	5,112	48.5	20,454	48.5	.979
<60	5,433	51.5	21,726	51.5	.
Sex					
Male	6,603	62.6	26,413	62.6	.996
Female	3,942	37.4	15,767	37.4	.
Comorbidities					
Diabetes mellitus	3,335	31.6	13,210	31.3	.542
Congestive heart failure	1,378	13.1	5,310	12.6	.186
Chronic kidney disease	1,947	18.5	7,599	18.0	.285
Autoimmune diseases	767	7.3	2,886	6.8	.119
Cerebrovascular disease	1,758	16.7	6,965	16.5	.695
Coronary artery disease	266	2.5	873	2.1	.004
Malignant neoplasms	953	9.0	1,268	3.0	<.001
Degree of urbanization					
urban	5,511	52.3	22,040	52.3	.990
suburban	3,469	32.9	13,899	33.0	
rural	1,565	14.8	6,241	14.8	
Income group					
missing	0	0.0	3	0.0	.861
low income	4,948	46.9	19,791	46.9	
median income	3,891	36.9	15,563	36.9	
high income	1,706	16.2	6,823	16.2	
Follow-up years (median)	3.90 (1.26–7.30)		5.85 (3.21–8.53)		<.001

### Incidence Rate of Depressive Disorders

**Figure 1:**
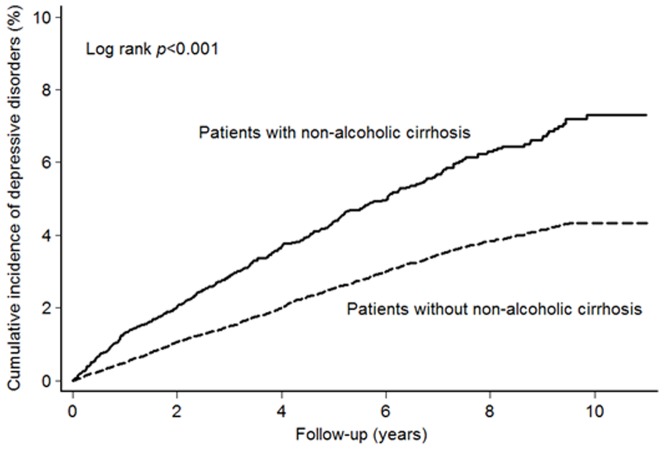
Cumulative Incidence of Depressive Disorders in Patients with and without Non-alcoholic Cirrhosis.

During the study period, 395 (3.75%) non-alcoholic cirrhotic patients and 1 183 (2.80%) control patients were diagnosed with depressive disorders. The incidence risk ratio (IRR) of depressive disorders between non-alcoholic cirrhotic patients and control patients was 1.76 (95% CI, 1.57–1.98, *P*<.001). When stratified by gender and age, the IRR of depressive disorders was higher in the non-alcoholic cirrhotic patients than in the control patients. The results are shown in Table 2. In Fig. 1, we present the cumulative incidence of depressive disorders during the follow-up period in the non-alcoholic cirrhotic and control patients.

**Table 2:** Incidence of Depressive Disorders in Patients with and without Non-alcoholic Cirrhosis.

	Patients with non-alcoholic cirrhosis		Patients without non-alcoholic cirrhosis		Risk ratio (95% CI)	*P* value
	No. of Depressive disorders	Per 1 000 person-years	No. of Depressive disorders	Per 1 000 person-years		
Total	395	8.4	1,183	4.8	1.76 (1.57–1.98)	<.001
Age						
≥60	132	7.4	560	5.3	1.39 (1.14–1.69)	<.001
<60	263	9.1	623	4.4	2.07 (1.78–2.39)	<.001
Sex						
Male	220	7.8	629	4.1	1.91 (1.63–2.24)	<.001
Female	175	9.4	554	6.0	1.57 (1.32–1.87)	<.001

CI indicates confidence interval

### Non-Alcoholic Cirrhosis on Risks of Clinical Depression

After adjusting for age, sex, comorbidities, urbanization, and monthly income, the hazard ratio (HR) for developing depressive disorders during the follow-up period was 1.75 times (95% CI, 1.56–1.96, *P*<.001) greater for the non-alcoholic cirrhotic patients than for the control patients (Table 3).

**Table 3:** Analyses of Risk Factors for Depressive Disorders in Patients with and without Non-alcoholic Cirrhosis.

Predictive variables	Univariate analysis		Multivariate analysis	
	HR (95% CI)	*P* value	HR (95% CI)	*P* value
non-alcoholic cirrhosis	1.75 (1.56–1.96)	<.001	1.75 (1.56–1.96)	<.001
Age<60	0.95 (0.86–1.05)	.347		
Sex (female)	1.41 (1.28–1.56)	<.001	1.36 (1.23–1.50)	<.001
Comorbidities				
Diabetes mellitus	1.38 (1.24–1.53)	<.001	1.26 (1.14–1.41)	<.001
Congestive heart failure	1.13 (0.97–1.33)	.126		
Chronic kidney disease	1.41 (1.24–1.59)	<.001	1.30 (1.14–1.47)	<.001
Autoimmune diseases	1.48 (1.24–1.76)	<.001	1.31 (1.10–1.56)	.003
Cerebrovascular disease	1.32 (1.15–1.51)	<.001	1.18 (1.03–1.35)	.021
Coronary artery disease	1.15 (0.79–1.67)	.476		
Malignant neoplasms	1.05 (0.77–1.42)	.770		
Degree of urbanization				
urban	referent		referent	
suburban	0.79 (0.70–0.88)	<.001	0.79 (0.70–0.88)	<.001
rural	1.00 (0.87–1.16)	.993	1.02 (0.88–1.18)	.787
Income group				
low income	referent		referent	
median income	0.89 (0.80–1.00)	.041	0.87 (0.78–0.97)	.011
high income	0.98 (0.85–1.12)	.764	1.01 (0.88–1.16)	.907

HR indicates hazard ratio; CI indicates confidence interval.

### Risks Factors for Depression in Non-Alcoholic Cirrhotic Patients

In Table 4, we applied univariate analysis to predict the development of depressive disorders in the non-alcoholic cirrhotic cohort, and the results demonstrated that an age less than 60 years old (HR  = 1.30, 95% CI, 1.05–1.61, *P* = .014), the female sex (HR  = 1.22, 95% CI, 1.00–1.49, *P* = .052), and suburban residency (HR  = 0.73, 95% CI, 0.58–0.92, *P* = .008) are significant prognostic factors. The multivariate analysis confirmed that an age less than 60 years old (HR  = 1.31, 95% CI, 1.06–1.62, *P* = .014), and the female sex (HR  = 1.25, 95% CI, 1.02–1.52, *P* = .031) were independent risk factors for depressive disorder in the non-alcoholic cirrhotic patients. Suburban residency (HR  = 0.75, 95% CI, 0.60–0.95, *P* = .015) was an independent protective factor for depressive disorder in the non-alcoholic cirrhotic patients.

**Table 4:** Analyses of Risk factors for Depressive Disorders in Non-alcoholic Cirrhotic Patients.

Predictive variables	Univariate analysis		Multivariate analysis	
	HR (95% CI)	*P* value	HR (95% CI)	*P* value
Age<60	1.30 (1.05–1.61)	.014	1.31 (1.06–1.62)	.014
Sex (Female)	1.22 (1.00–1.49)	.052	1.25 (1.02–1.52)	.031
Comorbidities				
Diabetes mellitus	1.14 (0.92–1.41)	.240		
Congestive heart failure	1.09 (0.78–1.52)	.614		
Chronic kidney disease	1.19 (0.92–1.55)	.187		
Autoimmune diseases	1.29 (0.91–1.83)	.147		
Cerebrovascular disease	0.85 (0.62–1.16)	.307		
Coronary artery disease	1.48 (0.79–2.78)	.220		
Malignant neoplasms	0.69 (0.40–1.20)	.184		
Degree of urbanization				
urban	referent		referent	
suburban	0.73 (0.58–0.92)	.008	0.75 (0.60–0.95)	.015
rural	0.91 (0.68–1.22)	.540	0.96 (0.71–1.29)	.782
Income group				
low income	referent			
median income	0.87 (0.70–1.08)	.213		
high income	0.89 (0.67–1.17)	.404		

HR indicates hazard ratio; CI indicates confidence interval.

## Discussion

The key findings of our study are that (1) the incidence rate of depressive disorders in non-alcoholic cirrhotic patients was 8.4 per 1000 person-year; (2) the risk of clinical depressive disorders was higher when patients had non-alcoholic cirrhosis (HR  = 1.75); (3) an age less than 60 years old (HR  = 1.30) and the female sex (HR  = 1.22) were independent risk factors for depressive disorders in non-alcoholic cirrhotic patients.

The strengths of this study are the large sample size, the long follow-up period, and the diagnosis of clinical depressive disorders by specialists. In addition, our study design involved an unbiased participant selection process. Because participation in the NHI is mandatory and all residents of Taiwan can access low-cost health care, referral bias is low and follow-up compliance is high.

This study is unique because we included only non-alcoholic cirrhotic patients (ICD-9-CM codes: 571.5 and 571.6) and excluded those diagnosed with alcohol-related disorders. Several studies have demonstrated that alcohol users were highly associated with psychiatric disorders, especially depressive disorders [9]–[14]. In alcoholic cirrhotic patients or non-alcoholic cirrhotic patients diagnosed with alcohol-related disorders, it is difficult to establish whether depression is related to alcohol or cirrhosis. Therefore, we designed this study to include only non-alcoholic cirrhotic patients.

Consistent with the results of previous studies [2]–[5], we found that the risk of depression in cirrhotic patients was higher than that in the non-cirrhotic control patients. Chronic inflammation is one of the possible causes. Cirrhosis is a chronic inflammatory disease, and chronic inflammation has been reported to increase the risk of depression [20]–[23]. A study based in China indicated that the psychological distress in cirrhotic patients, such as depression, anxiety, and insomnia, were correlated specifically to increased levels of aspartate aminotransferase, which is a vital mediator of inflammatory processes, which indicates the role of inflammation in the cirrhosis comorbid with depression [8]. In addition, impaired serotoninergic neurotransmission in cirrhotic patients may contribute to the underlying pathology [24]–[26]. The liver plays an important role in serotonin metabolism, being involved in its formation and inactivation. A Russian study revealed that blood serotonin content decreased in cirrhotic patients diagnosed with pronounced depressive disorders [22], [27]. Disturbance in melatonin homeostasis was also observed in patients with cirrhosis [24], [28]. Disarrangements in the rhythm and amplitude of melatonin secretion may account for symptomatic disturbances to sleep and mood in cirrhotic patients [29]–[31]. Furthermore, the symptoms of cirrhosis such as easy fatigue may impair patients' quality of life and influence patients' perception of health status, and make the patients more prone to depression [4], [32].

Previous studies demonstrated that a depressed mood was noted in approximately 50% of cirrhotic patients [3]–[5]. This prevalence rate was much higher than the rate calculated in this study. There are two possible reasons for these two discrete findings. First, in former studies, patients with alcoholic cirrhosis were included. As mentioned previously, depressive disorders are highly prevalent in alcohol users [9]–[14]. Second, rating scales, such as the Beck depression inventory, rather than diagnosis by a physician, were used for evaluation of depression in those studies. People who were recorded as depressed on rating scales may not meet the diagnostic criteria for depressive disorders. However, depressive disorders in cirrhotic patients may be underdiagnosed in our study because symptoms of depression may mimic symptoms of cirrhosis. Fatigue, loss of energy, sleep disturbance, and poor appetite are frequently experienced by cirrhotic patients, and these symptoms are also symptoms of depression. Physicians may have viewed cirrhotic patients' depressive symptoms as physical discomforts caused by cirrhosis and overlooked these symptoms, rather than viewing these symptoms as warning signs of depression [22].

We found that the incidence of depressive disorders was greater among female cirrhotic patients, and that the female sex is an independent risk factor for the development of depressive disorders in cirrhotic patients. Our findings are consistent with the results of a previous study that found that a higher percentage of women belonged to the depressed group than to the non-depressed group of cirrhotic patients [33]. In addition, an age less than 60 years is also an independent risk factor for the development of depressive disorder in cirrhotic patients according to our findings. It was possible that the amplitude of functional impairment caused by cirrhosis may be more prominent in younger cirrhotic patients than in older cirrhotic patients and that this made younger cirrhotic patients prone to depression[32].

Our findings have certain limitations. Information regarding the family history of psychiatric disorders [34], [35], lifestyle factors [36], self-perceived health, disability [37], and environmental factors (life stresses, such as recent major life event, domestic violence, childhood trauma, and social support) [38]–[41] are not included in the NHIRD, all of which may be associated with the risk of depressive disorders. In addition, the severity of cirrhosis could not be ascertained. Whether the severity of cirrhosis influences the risk of developing depressive disorders warrants further study, despite previous studies indicating that depressive symptoms in cirrhotic patients are not related to the severity of cirrhosis [5], [8]. Furthermore, physicians may view cirrhotic patients' depressive symptoms as physical discomforts caused by cirrhosis and overlook these symptoms. The incidence of depressive disorders in our study may be underestimated.

This population-based retrospective cohort study demonstrated that the risk of developing depressive disorders is higher among non-alcoholic cirrhotic patients than among patients without cirrhosis, and that non-alcoholic cirrhosis is an independent risk factor for depressive disorders. An age less than 60 years and the female are also independent risk factors for depressive disorders in non-alcoholic cirrhotic patients. Because depression was related to impairing the quality of life of cirrhotic patients, and depression is a modifiable illness that is amenable to treatment, symptoms of depression should be sought in patients with cirrhosis. Future population-based prospective studies are needed to further investigate the association between cirrhosis and the risk of developing depressive disorders.
